# Exploring the impacts of COVID-19 on Alberta correctional workers

**DOI:** 10.1016/j.heliyon.2024.e30213

**Published:** 2024-04-26

**Authors:** Matthew S. Johnston, Rosemary Ricciardelli, Ryan Coulling

**Affiliations:** a Memorial University of Newfoundland, St John's, NL, Canada; b Providence University College and Theological Seminary, Otterburne, MB, R0A 1G0, Canada

**Keywords:** COVID-19, Correctional workers, Incarcerated people, Occupational stress, Policy

## Abstract

COVID-19 and the subsequent public health responses disrupted the routines and lives of people globally. The impact was felt by correctional workers who navigated rapidly changing public health policies and many disruptions to operations within both institutional and community correctional services. In the current study, we unpack qualitative findings emerging from an online mental health and well-being survey, during COVID-19, of 571 correctional workers employed in the Canadian province of Alberta. Results emphasize how correctional work was strained by the on-set of the COVID-19 pandemic, creating other risks and vulnerabilities for both staff and incarcerated people. Respondents highlighted impacts to their workload, routine, personal and institutional security, relationships with colleagues and incarcerated people, and their competing perspectives on the enforcement and ethics of ensuing public health measures intended to contain the spread of the virus. We discuss the empirical implications of these findings and areas for future research post pandemic.

## Highlights

• The COVID-19 pandemic disrupted correctional worker routines, policies, and workload.
• Correctional workers have diverse perspectives on public health management.
• The COVID-19 pandemic created risks and vulnerabilities for incarcerated people.

## 1 Introduction

During the COVID-19 pandemic, prisons were identified as high-risk sites for the rapid spread of the virus “due to intersecting factors that included the health and social vulnerability of their residents, (over)crowding living arrangements that made physical distancing difficult or impossible, shared dining and sanitation spaces (e.g., bathrooms and showers), and the daily movement of large numbers of people (i.e., staff and visitors) into and out of prisons” [[1], [2], [3], [4], [5], [6]]. Other institutional factors exacerbating the virus contagion in prisons included poor ventilation and the strict control of hygiene items such as soap, cleaning supplies, and hand sanitizers [7].

The risks and vulnerabilities prison spaces create are shared by both incarcerated people and institutional correctional workers, who are constantly exposed to the challenges of the physical environment and operational realities of prisons such as prisoner violence, uncontrolled physical contact during altercations, and exposure during physical or medical examinations [8,9]. Recent research found institutional correctional workers in particular faced a heightened risk of contracting COVID-19 when compared with the general population [10,11] or experienced psychological distress/interpersonal difficulties from the increased health risks and demanding nature of correctional work exacerbated by the pandemic [7,12,13].

Prison systems across international jurisdictions responded to the dangers and consequences of the COVID-19 pandemic by adopting various risk mitigation strategies, including mask and personal protective equipment (PPE) mandates for staff; regular testing of staff and incarcerated people; limiting prison residents’ time outside of their cells; terminating visitation and programming; decreasing staffing levels; and reducing the number of people in custody [1,5,14,15]. Curbing the implementation of these emergency directives were calls from researchers to consider the mental health and well-being of incarcerated people, warning of the effects of isolation on incarcerated people with pre-existing mental health disorders or additional stressors caused by visitation restriction, decreased outdoor time and recreation, unavailable programming, and social disconnectedness in prison settings [16,17].

In collaboration with Alberta Division of Correctional Services, we undertook a mental health and well-being survey to identify the factors that were most impactful on correctional workers’ experiences with mental health and well-being during COVID-19. The qualitative findings emerging from the current study emphasize how correctional work in Alberta, Canada was strained by the on-set of the COVID-19 pandemic and the rapid organizational changes to policy and procedures that followed to protect public health. Given the lack of research tied to the impacts of the precedent setting global pandemic and associated public health measures, the strengths of the study include the need to fill an evidence gap in how to prepare for future pandemics through analyses of lived experiences. Correctional workers highlight the impacts to their workload, routine, personal and institutional security, relationships with colleagues and incarcerated people, as well as their competing perspectives on the enforcement and ethics of ensuing public health measures to contain the spread of the virus. We discuss the empirical implications of these findings and suggest how correctional services—operating both in the community and in institutions—can move forward amidst diverse experiences, perspectives, and social divisions.

## 2 Literature review

In their qualitative study of 96 institutional parole officers conducted after Canada's “first wave” of COVID-19 infections, Norman and Ricciardelli found Canadian correctional work was affected—both positively and negatively—by shifting workloads, routines, and responsibilities; increased workloads due to efforts to reduce the number of incarcerated people; and the navigation of new forms of occupational stress, risk, need, and uncertainty [1]. Specifically, they found remote work increased institutional parole officers' productivity since they could concentrate better on report writing and applications when removed from the chaotic prison environment; yet this finding was nuanced by the overlap of work and personal space which, for some participants, reduced the boundaries necessary for maintaining work-life balance [1]. Participants in this study also spoke about the negative impacts decreased communication with clients had on their capacity to build relationships and meet clients' emotional, psychological, social, or programming needs [1]. They reported inadequate safety protocols, spatial arrangements, and the behaviour of incarcerated people as sources of risk to both personal and public health [1]. Further, they perceived worker safety concerns were being undermined by a lack of consistent enforcement of COVID-19 protocols and de-prioritized by management [1].

Canadian federal correctional officers in Schultz and Ricciardelli's study on worker perspectives on COVID-19 restrictions, legitimacy, and prison order describe how inconsistent messaging around COVID-19 protocols hindered trust-relationships with incarcerated people [18]. The authors found individual and competing definitions of safety informed day-to-day prison routines, as communication deficits and shortcomings led to a legitimacy crisis among officers who felt prison policies “left officers and incarcerated people in a near-constant state of conflict”– especially when incarcerated people viewed officer-enforced COVID-19 restrictions as “capricious exercises of power” [18]. These findings nuance other research framing correctional officer responses to the pandemic through conservative politics and vaccine resistance [19], as officers in the study varied in how they interpreted COVID-19 restrictions and mandates, often through a lens that carefully weighed their own safety against the day-to-day realties of prison work and organizationally mandated priorities [18].

In research examining how 21 correctional officers working in the southeastern United States experienced COVID-19, one study found the pandemic significantly disrupted institutional operations, exacerbated health concerns for officers, and created a sense of confusion and annoyance among officers over the “lack of continuity and logic” in procedures designed to contain virus spread [20]. More specifically, officers reported being frustrated over how physical constraints imposed by PPE would create behavioural problems across the institution, anger toward perceived unfair disciplinary practices, and sympathy to incarcerated people's frustration over the additional restrictions imposed on their bodies and movement [20].

Black, female correctional officers in a United States study reported higher levels of burnout, exhaustion, and workload because of the strenuous work conditions brought on by the pandemic, and a lack of available mental health services from the Department of Corrections at Rikers Island Correctional Facility [21]. In another United States study of 78 correctional sites in Pennsylvania, Maryland, West Virginia, and New York, researchers found, during the pandemic, approximately 48 % of correctional healthcare workers and 32 % of correctional officers reported mild to severe depressive symptoms; 47 % of correctional healthcare workers and 57 % of correctional officers reported symptoms of burnout; and 50 % of correctional healthcare workers and 45 % of correctional officers reported posttraumatic stress symptoms [7].

In a similar vein, one Master's-level study found that Canadian correctional officers experienced elevated stress because of disruptions to correctional system operations and heightened safety concerns [22]. While another study also reported Canadian public safety personnel believed the pandemic brought on new challenges pertaining to childcare, finances, work, emotional and social health, and access to mental healthcare [23], few participants in this study described these impacts as ‘severe’ and many indicated they were not significantly impacted by the pandemic [23]. In other international research, research found correctional officers working for the Nigerian Correctional Service scored high (mean knowledge score 19.34 out of 25) during pre- and post-test assessments of their COVID-19 knowledge, attitudes, and preventative practices, and that awareness training significantly improved participants' scores, thus emphasizing the necessity of staff education [24]. Another study of 375 randomly sampled male correctional officers working at five South Korean correctional facilities identified participant job stress was negatively correlated with COVID-19 knowledge, attitudes, and practices [25]. They concluded that “efforts to increase the level of knowledge about COVID-19 will help the employees to manage their job stress, and this will improve job quality” [25].

Together, emerging research on the effects of COVID-19 on correctional staff emphasizes the complexity and differences in how correctional workers across jurisdictions have interpreted and experienced the pandemic. Yet less is known about how Canadian provincial and territorial correctional workers handled the policy, social, and material shifts during the COVID-19 pandemic and reflected on these system changes through various experiential or ethical perspectives – a finding the current study seeks to further unpack qualitatively through the lens of Alberta correctional workers.

## 3 Method

### 3.1 Materials

Provincial correctional workers in the Canadian province of Alberta (*N* = 571) were invited to complete an online survey in early 2022. The broader mental health and well-being survey included established self-report screening measures for several mental health disorders, as well as questions pertaining to demographics, history of potentially psychologically traumatic event exposures (including those specific to working in correctional services), and other content focused on workplace experiences, personal relationships, and well-being. Questions included a mixture of both structured and open-ended options, to allow respondents the opportunity to elaborate on the various contexts and personal or workplace events that shaped their occupational and mental health experiences. While the survey did not include specific open-ended questions pertaining to COVID-19, the impacts of the pandemic emerged as a theme across responses to several questions, including perspectives, ideas, and experiences around public health measures, working from home, personal protective equipment, vaccination, workload, client diversion, operational impacts, effects on communication and client contact, frontline and management disconnection, and effects on personal and institutional security.

### 3.2 Procedure

Informed consent language approved by the research ethics boards at the University of Regina (file #2017-098) and Memorial University of Newfoundland (file #20201330-EX) was included in the first pages of the anonymous, voluntary, and confidential survey. To access and complete the survey, participants were required to first read and agree to the provided informed consent language, which detailed the purpose of the survey, the potential benefits and risks, and the estimated time required for completion. Survey responses were hosted on a secure server, via Qualtrics. Qualtrics uses Transport Layer Security (TLS) encryption (i.e., HTTPS) technology that protects user information with both server authentication and data encryption. Qualtrics does not transmit to other parties any data recorded during use and does not store data in the United States (i.e., navigating the mandates of *The Patriot Act* which could violate confidentiality).

Prior to commencing the survey, participants were asked to create a unique login access code, referred to as a nondescript unique response ID (participants’ IP address information is removed from the data set and deleted from the server following data collection). The code allowed participants to login into the survey and input survey responses from any computer as well as complete the survey over multiple sittings, if desired. Participant identities were further protected by making recovery of a lost code impossible; as such, participants who lost their codes and wanted to continue participating would have had to restart the survey. Participants could refuse or choose to exit the survey at any time without consequence. The research team signed and abided by non-disclosure agreements to ensure all survey information was confidential, and the anonymous data collected from the survey were used to conduct the analyses with sensitivity and care.

The median time to complete the survey was 57 min; however, there was considerable variation due to survey logic skip patterns. Respondents were able to skip sections based on responses to screening questions and associated skip logic. Relevant to the current study, variation in the length of responses to open-ended questions also impacted completion times.

### 3.3 Participants

A total of 571 respondents logged into the survey, but 553 (96.8 %) answered the question on occupational category near the beginning of the survey. Additionally, 537 (94.0 %) respondents who had logged into the survey responded to the sociodemographic questions at the beginning of the survey (i.e., some did not proceed far enough into the survey to complete the preliminary sections). The nature of this response rate means sample sizes of respondents across questions will vary. Of the respondents who responded to the sociodemographic questions in the broader survey, 43.9 % identified as male and 56.1 % identified as female. The average age was 41.5 years (median = 41.0). Most respondents reported being married or in a common law relationship (63.9 %), but 21.6 % were single, 11.4 % were divorced/separated/widowed, and 3 % reported being remarried. Most respondents reported completing a four-year university/college program or higher (50.5 %), while many reported some post-secondary education less than a four-year degree (41.6 %) and some reported high school education or less (8.0 %). Respondents reported an average of 14.1 (median = 11.0) years of correctional service.

Operational (custody), which included correctional officers and persons working to provide safety, control, and care in institutions, represented 41.6 % (*n* = 230) of the sample. Other occupational groups include: operational (community; e.g., probation officers) which constitutes 30.7 % (*n* = 170) of the sample, management and administrative (custody) is 14.5 % (*n* = 80); management and administrative (community) is 8.7 % (*n* = 48), and administrative (head office) is 4.5 % (*n* = 25) of the sample (see Table 1 for more information on participant demographics).

**Table 1:** Sample demographic information of Alberta correctional workers (*N* = 571).

Demographics	% (*n*)
Total Sample	
Sex (*n* = 526)	
Male	43.9 (231)
Female	56.1 (295)
Age (*n* = 521)	
20-29	11.7 (61)
30-39	355 (185)
40-49	29.9 (156)
50-59	17.3 (90)
60 and older	5.6 (29)
Marital status (*n* = 527)	
Single	21.6 (114)
Married/Common-law	63.9 (337)
Separated/Divorced	10.6 (56)
Remarried	3.0 (16)
Education level (*n* = 515)	
High school graduate or less	8.0 (41)
Some post-secondary school	41.6 (214)
University/college degree or higher	50.5 (260)
Years of service (*n* = 520)	
Less than 4 years	11.2 (58)
4–9 years	28.5 (148)
10–15 years	27.7 (144)
More than 15 years	32.7 (170)
Urban vs rural work location (*n* = 537)	
Urban	97.2 (522)
Rural	2.8 (15)
Occupational Group (*n* = 553)	
Operational (Custody)	41.6 (230)
Operational (Community)	30.7 (170)
Administrative (Custody)	14.5 (80)
Administrative (Community)	8.7 (48)
Administrative (CSD Head Office)	4.5 (25)

### 3.4 Analysis

Our analytic process was thematically inductive [26] given that we employed a constructed *semi-grounded* emergent theme approach to data coding, analysis, and organization [27,28], meaning we did not pre-emptively discern what themes would emerge from the data and we framed the study according the data revealed empirically while not creating any theory [29]. The second author (principal investigator), third author, and other research assistants analyzed data by coding each participants' words first into ‘parent’ (e.g., primary themes) and then ‘child’ (e.g., secondary themes) and ‘grandchild’ (e.g., tertiary themes) nodes based on themes emerging in QSR NVivo software. The first author then read through and reviewed the coding, and finalized categorized predominant themes found across all responses. The data emerging across questions and responses were combined into one file for ease of analysis. The quotes from respondents we present in the results highlight respondent voices and have undergone minor edits to assist with readability, flow, and to correct grammatical and spelling errors, making sure to never compromise interpretations, meaning, or tone. To improve accuracy, we have opted to include the percentages of themes found across open-ended responses. A theme was operationalized as multiple participants reporting similar interpretations, experiences, or ideas; said differently, quotations from multiple respondents coded in a theme became an emergent theme.

## 4 Results

Respondents described throughout the survey how COVID-19 affected their personal lives and occupational roles and duties. We analyzed the key themes found across survey responses, which included impacts and concerns pertaining to workload, contact with clients, communication, enforcement of public health measures, client diversion, personal and institutional security, working from home, and frontline and management disconnection.

### 4.1 Impacts on workload

A total of 35 (6.1 %) respondents across occupational groups described the impacts COVID-19 had on their workload, often marked by increased demands on correctional workers' time and an expansion of the scope of their duties and responsibilities: “Completely changing basic routine and duties, such as requiring staff to conduct extra rounds and medical rounds with our health care partner” (P377, Administrative Custody); “Constant policy and procedural changes are hard to keep up with” (P105, Administrative Custody); “I am worried about being short staffed if I or someone else has to be off to self-isolate or is ill. I'm worried that cover staff won't be provided” (P051, Probation Officer). In each of these three responses, correctional staff working in both institutional and community settings note how the abrupt and lasting changes and disruptions to their work environment were a source of stress, whether from the added healthcare responsibilities that accompany being a first-responder; the need to adapt to the volatile policy and procedures being implemented to respond to the evolving pandemic; or the extra burdens, fears, and dangers emerging alongside human resource shortages that become nearly impossible to mitigate with the constant presence of contagion.

Other respondents remarked more specifically on how the pandemic increased their workload by virtue of additional duties in conjunction with a decrease in client contact, the latter of which is integral to developing rapport and maintaining institutional cohesion: “Virtual court has had a huge impact on my responsibilities” (P105, Administrative Custody); “There is a lot more reporting required for COVID-19 cases within the facility. This puts an unfair pressure on administrative staff and caused a breakdown of communication between on-site AHS [Alberta Health Services] and corrections staff with the administrative staff” (P133, Administrative Custody); “I am unable to see my clients and know first-hand how they are really doing. It's harder to develop a rapport with people we don't see” (P051, Probation Officer). In the first quotation, P105 describes a change that occurred in their responsibilities with more clients having to access virtual court. In the second response, P133 notes how administrative staff too have been challenged by increasing demands of paperwork and reporting, which has also led to entanglements between frontline and administrative staff who, due to less communication between departments, may be un- or less aware of the working realities the pandemic triggered for each respective sector of correctional work. P051, a probation officer, then explains the challenges associated with rehabilitating and overseeing the care of incarcerated people with whom they have limited in-person contact due to COVID-19 public health measures.

P073, a correctional service worker, added further nuance to the impacts diminished client communication had on their working life with populations serving a custodial sentence: “My responsibilities have not changed, but it makes doing them harder. I'm no longer able to see clients in person. This reduces the effectiveness of my ability to help them over the phone.” Here, the correctional service worker laments their reduced capacity to “help” incarcerated people, as there is a sense that phone contact is not synonymous with in-person contact, where the absence of non-verbal cues and expressions may lead to less rapport and trust building. Another respondent, P024, who works in institutions, stated: “It has increased my workload as I have been tasked with providing activities for those on quarantine and isolation as well continu[ing] my regular case load”. This correctional service worker struggles to maintain balance managing their “regular” caseload while providing much-needed recreation for the many incarcerated people who find themselves in quarantine and isolation in what are already restrictive environments.

The extra duties and responsibilities also cost correctional institutions in terms of finances and human resources: “I feel we [are] understaffed and put under extreme budget pressure at the same time” (P032, Probation Officer). One participant, P104 (Correctional Service Worker), discussed in depth how staff shortages affected their workplace: “During an outbreak, our staff was severely limited. I worked 80 h a week during this time to alleviate staffing issues. Now that the outbreak is over, I wear PPE all shift, maintain social distancing, and conduct my duties regularly.” Here, the respondent worked hard and unsustainable hours to make up for the loss in staffing, while adhering to the sometimes-taxing public health requirements meant to contain the spread of COVID-19 in their institution.

Respondents, especially those working in institutions, also spoke about the difficulties managing their workload with a reduced population, as many incarcerated people were diverted to the community in an effort to reduce the spread of COVID-19: “It has made the population I am dealing with smaller, but restricting inmates' exercise, therefore making them more angry and thus, making my job harder” (P145, Correctional Officer); “Higher workload, expectations to keep Centre population at a good level all while releasing as many inmates as possible on fine option … This has made it a difficult balancing act, especially since Sheriffs have lowered their transportation capacity to 3 from 10 and are still only having one run a week to our Centre” (P091, Correctional Service Worker). In the first quotation, P145 describes the burden placed on incarcerated people who, due to the pandemic, have less opportunities to exercise and therefore encounter more physical and mental stress, which correctional workers must do their best to mitigate all the while trying to balance the best interests of public health and safety. The second response from P091 indicates the hardship of trying to maintain client populations needs, especially when client transportation infrastructure and accompanying policies limited correctional workers’ capacity to release low-risk incarcerated people back into the community.

Inherent to the complexities of trying to manage increased job expectations is the stress associated with believing that few incarcerated people or members of the public fully understand the extra hardships faced by correctional staff trying to navigate their work during the pandemic: “Job pressures and all related duties have doubled. Lack of public support and threats of my department being sued by inmates alleging they were not protected enough from COVID while we put ourselves and families at risk for them is infuriating” (P409, Correctional Manager). The pandemic was hard on all actors involved in the correctional system, but this passage points to some of the divisions present between correctional workers, incarcerated people, and the ‘unsuspecting’ public whom some correctional workers believe do not appreciate the lengths staff have gone to mitigate or protect incarcerated people from the dangers and consequences of the pandemic [30].

### 4.2 Impacts from personal protective equipment (PPE) and vaccine requirements

Thirty-five (6.1 %) respondents commented on the physical and social impacts PPE and vaccine requirements had on their occupational duties and responsibilities; as such, responses were rife with fears surrounding the contraction of COVID-19. In addition, there were criticisms about the ethics of PPE and vaccination requirements and/or waning compliance; as well as worries about how PPE disrupts and hinders communication efforts between incarcerated people, probationers and correctional workers, especially during critical incidents. Two survey responses especially exemplified the divisions and differences found concerning how respondents approached COVID-19 policies and public health measures:

Yes, we are still told to see clients in our offices - high risk clients but we have PPE. I still am really concerned [because] the populations of clients I work with do not get vaccinated, believe COVID-19 is a hoax so don’t wear masks, remain at a safe distance from people, do not use cleaning techniques on surfaces. I am very anxious when people are in my office and just want to rush them out. (P318, Administrative Community)

The COVID policies are discriminatory, divisive, unfair, stressful and punitive. I am aware staff are required to test every 72 hours that increases their risk of exposure to any viruses. I have also heard the way that staff have pressed their personal beliefs on clients and spoken about them with other staff in a negative and derogatory manner with respect to clients choosing to exercise bodily autonomy. I have experienced situations where clients are not able to access resources due to them expressing bodily autonomy. This is happening to an already vulnerable and high needs/high risk population. It is not our role to encourage staff or clients to take a COVID injection, whether we have or have not taken it ourselves is irrelevant. That is inappropriate. We need to be impartial (as we are not experts in this area) and work with our clients where they are at. (P385, Probation Officer)

Participants, working in institutions or in the community, felt their safety at work was jeopardized; this included incarcerated people or probationers largely resisting vaccination and PPE because they did not believe COVID-19 was real or a serious danger. P318 describes the ensuing anxiety of trying to maintain and promote public health while working with clients who, though needing correctional workers’ support, were still engaging in behaviours P318 believes put their personal workplace safety—quite unnecessarily—at risk. This conundrum caused the correctional worker to “rush” their duties in an effort to minimize contact with clients.

P385, on the other hand, describes a workplace institutional culture marked by a seeming division and “unfair” practices and pressures to comply with COVID-19 policies and protocol. They believe clients were punished for exercising their bodily and medical autonomy to abstain from PPE requirements or vaccination – undoubtedly, these behaviours in widespread cumulation jeopardize institutional security and health, but this correctional worker is adamant most correctional staff are not in an objective position to “encourage” public health practices because they lack “expertise”. While P318 views the lack of client compliance as a workplace danger and safety risk, P385 sees—and even appreciates—client vulnerability as driving their personal decisions and thus emphasizes the need to meet “clients where they are at” and not react punitively.

Other respondents were clear about how both staff and client failure to abide by and enforce public health measures created occupational stress:

My anxiety would be through the roof if we had to be in the office. Going to the office is the only times I’m worried about getting sick because co-workers do not follow PPE protocol while there. (P144, Administrative Community)

I have been required to be more focused on enforcing PPE policies, as well as ensuring staff understand the reasons for the changes to policies regarding placement, PPE usage, and inmate management. (P130, Correctional Officer)

We have had to watch for the symptoms cause the offenders will try to hide because they don’t want to isolate. (P081, Correctional Officer)

The first quotation, from P144, emphasizes the fear, discomfort, and anxiety some correctional workers experienced because of a lack of compliance to COVID-19 policies. P130 then discusses the shift in occupational focus that occurred because of the need to educate and potentially re-educate staff about the constantly changing and evolving policies surrounding PPE, client placement, and management. The third quotation, from P081, describes an extra responsibility placed on correctional officers to seek out incarcerated people who may be hiding symptoms to avoid isolation, once again placing burden and health risks on everyone exposed to the virus, as well as social consequences for correctional workers who may be seen as antagonistic for trying to identify potentially sick and contagious incarcerated people.

Some respondents reiterated the impacts PPE requirements had on communication efforts with clients, who during an episode of distress or emergency need may not be able to communicate as effectively through masks, face shields, and so forth: “The amount and when we need to wear PPE has made it risky when responding to emergency situations. Security concern is an issue because we have been instructed to keep a distance. So, no pat down searches or searches of their cells have been done” (P081, Correctional Officer). P081 describes a withdrawal of security procedures in the interests of protecting public health, which this correctional officer believes increases staff risk and danger and decreases communication efficacy during emergency incidents.

In addition to not being able to search incarcerated people or their cells and being constricted by what they can see and observe through PPE, other respondents reported fatigue and exhaustion with COVID-19 protocol: “I hate wearing masks for 8 or more hours a day” (P034, Operations Custody); “It is uncomfortable for staff to have to wear masks all day” (P122, Correctional Manager). One respondent also reported an increase in the weaponization of bodily fluids, which PPE can only do so much to protect against:

We are required to wear mask and eyewear all of the time while on duty. When dealing with individuals suspected of having COVID-19, we wear gowns and attempt to keep our distance. However in our line of work this is mostly impossible. Offenders are unpredictable and often require us to have physical contact with them in order for functionality. Offenders often spit, throw bodily fluids at us and this has increased with COVID. (P054, Correctional Officer)

P054, a correctional officer, raises concern about the inherent nature of institutional correctional work which requires, at times, deeper and detailed communication practices with clients to achieve correctional results and maintain institutional order. As PPE can create challenges with communication, the correctional officer believes incidents and occurrences will transpire that make it “impossible” to abide to protocol, especially with a population that another respondent conceptualized as “on edge” (P063, Correctional Officer) because of the strains of the pandemic.

Another respondent, P407 (Correctional Officer), further described the paradox of trying to simultaneously preserve public health and public safety in a carceral setting:

PPE is great for a hospital setting but you cannot apply the same components used there to a correctional environment and expect the same result. For example, plastic eye wear is great eye protection in a medical setting but if they are not shatter proof in a correctional setting that is a great way to lose an eye in an altercation.

Underpinning much of data is how correctional environments—particularly institutions—present unique operational and political challenges to contain a pandemic through adequate policy and enforcement. Many participants were able to unpack these complexities in great depth, but often without clear answers, solutions, or directives about how to best maintain the balance and safety they strive to achieve in their work in the face of many impediments, challenges, and diverse/competing perspectives.

### 4.3 Other operational impacts

In total, 194 (34.0 %) respondents discussed other operational effects, disruptions, or controversies that surfaced as a result of COVID-19. Specifically, commentary on the effects of remote work (or lack thereof) were salient across data:

For the past 2 years I have successfully worked from home. I meet all deadlines and complete all tasks. (P370, Administrative Community)

It has been positive from the lens of Probation. We are working from home, huge benefit for me, but also clients don’t have to come in and they can report by telephone meaning a lot less people get breached for being poor and not having access to transportation. (P144, Probation Officer)

Now that everything is webex meetings, I suddenly have weeks with 5–6 meetings some weeks, mostly about nothing. All of a sudden everyone has instant access to meetings and people’s time without any consideration of other job-related duties. Huge added amount of stress!! (P322, Administrative Community)

More computer knowledge is required to set up a home office, not take files home. (P178, Administrative Community)

We still have to go to the office with many other Probation Officers present, which puts us all at risk. It is unnecessary as our clients are reporting by telephone. They still want us to do parts of our job that are hard if not difficult to do over the telephone with our clients. They moved to having some clients in our office, which puts us all at risk for COVID. I have family with autoimmune illnesses and me being around my work puts them at risk if proper protocols are not in place. (P401, Administrative Community)

In general, some community correctional workers expressed positive experiences from working remotely (i.e., reduced travel time; better client relationships; less disruption of clients’ lives from in-person meetings), while other participants experienced struggles with remote work (i.e., less productivity, technological disruptions, unpreparedness, isolation). Correctional workers in the community, such as P370 and P144, tended to favour the advantages of remote work, as remote work had a positive impact on the number of clients being breached due to travel accessibility and socio-economic challenges. This finding is nuanced, however, by the words of P401, who raised concern with being obligated to be present at their office at times despite their clients reporting by telephone; by proxy, this practice put their family members with autoimmune illnesses at risk.

Other respondents in both community and institutional settings described an increasing hostility toward management whom they perceived as being disconnected from the COVID-19 realities and burdens experienced by front-line staff:

Ongoing changes and seems like lack of support from upper [management]. They basically say it is what we need to do and shrug it off. No empathy with changes, workload and COVID. (P057, Operational Community)

Pandemic has brought out more stresses on top of what is already stressful. Upper management constantly changing therefore no ’direction’ to follow. Unanswered questions lead to anxiety. (P367, Correctional Officer)

While correctional managers were tasked and heavily pressured to implement policies and procedures that mitigated the impacts of the pandemic on all actors in the correctional system, what is critiqued here is a lack of follow-up with or “empathy” toward frontline staff who had to bear the brunt of the rapid, constantly shifting changes. P367, a correctional officer, continued to elaborate on these concerns:

Management more concerned about the inmates than they are about my safety. Management is killing us with the constantly changing of rules - rules that apply differently to different sets of inmates (based on lawyers) and management is constantly putting needs of inmates before staff. We run short staffed and are expected to do more with less. Staff are burning out.

P367 felt rules were being enforced with much discretion and discrepancy, often to the advantage of incarcerated people, which led to the correctional officer experiencing a sense of injustice. The burden is further intensified by staff burning out from the changes and being required to live up to the challenging expectations of their job, but with fewer resources. Select respondents critiqued the efficacy of and seemingly contradictory nature of some institutional COVID-19 policies:

Management demands some orders to make it appear they are doing more, when the orders do absolutely nothing. Essentially, managers are being dictators with absolutely no intelligent or otherwise reason for many things. Such as, we can no longer use our personal fitness room, even if working out alone; however, if we want to take inmates to their provided fitness area, we can work out with them. Or, mandatory wearing of a mask and glasses even if there is more than 20 feet and locked doors between anyone else, or simply just no person anywhere near you. (P076, Correctional Service Worker)

This respondent troubles how effective PPE and social distancing requirements are in a closed correctional setting, and once again, raises tension with the idea that incarcerated people have a different set of rules to follow than staff, despite their circumstances, needs, and status being quite different.

## 5 Discussion

Within carceral spaces, both institutional and community settings, COVID-19 created risk for correctional staff; not just of illness, but also of burnout, vulnerabilities, and fears [1,5,7,12,13,20]. Prison spaces were recognized early as congregate living spaces where COVID-19 was easily transmittable [[2], [3], [4],^6^], thus fear of contagion was real. As expressed in the current study, the vaccine hesitancy that prevailed among some incarcerated people and select correctional workers transferred to risk in prisons and also in the community, given probation officers continued to meet in closed spaces with those on their caseload who may have refused vaccination. Thus, the impacts of COVID-19 extend far beyond prisons, affecting criminalized populations and those responsible for their supervision in diverse and multiple ways.

More specifically, policies and procedures implemented across Alberta correctional services meant to mitigate the very real consequences of a pandemic created other risks. The PPE, alongside other public health measures, at times worked against correctional workers, creating blind spots, impairing communication with incarcerated people, and generally compromising safety. In this sense, measures intended to protect also increased vulnerabilities and generated new variants of harms. Studies launched at the outset of the COVID-19 pandemic found these emerging harms and vulnerabilities were exacerbated by communication breakdowns among staff and inconsistent organizational messaging [1,18]. The current study, which captures the perspectives and realities of correctional workers in early 2022, at a time when the pandemic responses and challenges had taken root in working life, points to how competing perspectives on how to best manage the pandemic (and the organizational divisions that followed) were nonetheless sustained.

We found COVID-19 increased the occupational burden associated with correctional work – in prisons and in the community – which is a finding that resonates with other literature highlighting the mental health plight of correctional workers during the pandemic [21]. Staff shortages, due to leaves of absences after contracting COVID-19 or as a preventative measure, exacerbated already trying workloads for all. New occupational duties arose, for instance contract tracing, vaccine distribution, supporting virtual court, and navigating remote work, to name only a few, and correctional workers lamented the strained relationship with incarcerated people and probationers that resulted from public health measures directed at correctional services. Administrative burdens rose alongside increases in applied duties and potential liabilities, which created vulnerabilities that were already heightened due to fear of contagion. A clear concern emerged around organizational communication challenges, where public health measures were ever changing, resulting in impaired abilities for managers to communicate and enforce new policies—for instance, a protocol could be communicated and changed minutes later due to new information. The communication gaps not only provided challenge but created distrust, particularly between people in managerial positions and those working under them.

The inability, in community correctional services, to meet probationers in person was also a challenge, impeding rapport building but also making sense of their circumstances and possible needs (as well as risks) impossible. In prisons, absent contact and visitation as well as PPE impeded rapport building at a time when loneliness and social disconnectedness among incarcerated people prevailed [16,17], given the lockdown and isolation imposed by COVID-19. This finding was exacerbated by staff shortages as being understaffed with an increased workload in turn decreased available time for communication and performing the arguably central mission of correctional work – the rehabilitation of incarcerated people or those serving sentences in the community. Moreover, in community correctional services, decarceration strategies for lower-risk incarcerated people increased community correctional workers’ workloads [1], as more people were requiring community supervision, but in ways that were rather offset by public health measures.

The push to vaccinate all actors inside the prison or in community, coupled with vaccine hesitancy, created new complications. Not all employees were comfortable with vaccinations but were nonetheless mandated, as was the case with many other professions in Canada, to be vaccinated to continue their employment. Moreover, incarcerated people were not all willing to comply with the mandate. Some staff felt encouraging compliance was outside their expertise and some were uncomfortable doing so – they are not scientists or healthcare professionals – but the expectation to push for vaccination became an occupational reality. The notion of “meeting clients where they are” in terms of ensuring they are comfortable with vaccination, informed about options, and ready to consider the vaccine was expressed – but the questions then are: how would that be possible and what staffing levels are necessary to ensure such practices are feasible, given the already strained human resources of Canadian prisons, coupled with increased responsibilities?

Nevertheless, some participants expressed how a lack of vaccination increased risk for staff and other incarcerated people, creating heightened vulnerabilities for contagion. This theme extended to community correctional services, as an unvaccinated incarcerated individual then became an unvaccinated probationer. Lack of compliance to public health measures, including vaccination, created anxiety, fear, and discomfort for many. This reality was also elevated by incarcerated people electing to hide symptoms of COVID-19 to avoid isolation, which also increased occupational burdens as the occupation entailed testing and surveying incarcerated people for signs and symptoms of infections, which is clearly beyond the mandate of any correctional occupational role. The tension, though, remains as the pandemic may be declared over but COVID-19 is still a concern – as of May 03, 2023, the World Health Organization (WHO) reports that, globally, COVID-19 had caused at least 6,921,614 deaths [31].

COVID-19 and public health measures remain in Canadian prisons, lockdowns still happen, and isolation continues – it is a congregate living space that requires hypervigilance to COVID-19. So how do we address the needs of incarcerated people, probationers, and all correctional staff while still ensuring people are protected and safe – psychologically, physically, and socially? Policies are constantly changing and arguably inconsistent between jurisdictions in Canada—including for good reason (varying rates of infection, population density, etc.). Thus, it is difficult to make recommendations based on a study across jurisdictions, where forms of governance are different, and the impacts of COVID-19 are discursive. The highly variable policies across jurisdictions and those policies that change within jurisdictions due to the shifting nature of COVID-19 and health responses also point to an “unsettled period” [32]. Further, research should investigate how correctional workers seek to inform a “settled” cultural and operational toolkit [33]. Additionally, future research ought to further unpack the nuance of COVID-19 impacts across different correctional professionals – particularly among managers and nurses. The current study hints to how managers experience unique challenges tied to communication and points to a need to explore how nurses, because of their frontline role, navigate public health measures in carceral spaces.

The current study is not without limitations. First, the data comprise open-ended survey responses and thus we did not have an opportunity to probe respondents for additional information. Second, qualitative data, however comprehensive, requires much caution with any attempt at generalizability. Additional limitations include the self-selection that underpinned participation and the fact that participation was likely impacted by the pandemic and related public health measures.

## 6 Conclusion

COVID-19 and public health responses disrupted the routines and lives of everyone. This was also true for correctional workers in Alberta, Canada, who, while dealing with the disruption to their personal lives, also had to navigate the operational and organizational effects and aftermath within correctional services. These effects included increased workloads as they navigated the entanglements of implementing public health measures and the burden of taking on additional responsibilities. In essence, Alberta correctional workers were trying to simultaneously mitigate the risks of COVID-19 as well as the risks inherent to the corrections profession, which created stress and sometimes, we argue, a form of moral distress or dilemma.

Further, the rapidly changing policies created communication barriers between departments and correctional workers. In addition to the added workload, anxiety was reported to increase as some processes to maintain and promote public health put clients, correctional workers, or both at risk. Some correctional workers believed COVID-19 rules were being enforced discretionally for incarcerated people, which created a sense of injustice. Finally, responses to remote work were mixed. Some community correctional workers expressed positive experiences, while other participants experienced struggles with remote work.

Ultimately, the response to COVID-19 within correctional services—and perspectives on such responses—were varied in the Canadian province of Alberta. Unsurprisingly, for correctional workers who often rely on regimented best practices to ensure the care and safety of themselves, their co-workers, and incarcerated people, the divisions in perspectives seem to have created added stress. Moving forward, there is a need to find measures that attend to public health and the safety of incarcerated people and correctional workers – measures we hope may be better informed through staff perspectives.

## Ethics declarations

This study was reviewed and approved by research ethics boards at the University of Regina (file #2017-098) and Memorial University of Newfoundland (file #20201330-EX). All participants provided informed consent to participate in the study.

## Data availability statement

Due to privacy and confidentiality reasons, the data that have been used are confidential.

## CRediT authorship contribution statement

**Matthew S. Johnston:** Writing – review & editing, Writing – original draft, Methodology, Formal analysis, Conceptualization. **Rosemary Ricciardelli:** Writing – review & editing, Writing – original draft, Supervision, Project administration, Methodology, Investigation, Funding acquisition, Formal analysis, Data curation, Conceptualization. **Ryan Coulling:** Writing – review & editing, Writing – original draft, Methodology, Formal analysis.

## Declaration of competing interest

The authors declare that they have no known competing financial interests or personal relationships that could have appeared to influence the work reported in this paper.
